# Overcoming barriers to ‘flip’: building teacher’s capacity for the adoption of flipped classroom in Hong Kong secondary schools

**DOI:** 10.1186/s41039-017-0047-7

**Published:** 2017-01-25

**Authors:** Tianchong Wang

**Affiliations:** Faculty of Education, The Chinese University of Hong Kong, Hong Kong SAR, China

**Keywords:** Capacity Building, Secondary School Teacher, Public Secondary School, Teaching Paradigm, Flip Classroom Model

## Abstract

Flipped classroom teaching has become a significant trend in education in recent years, but there remain significant challenges in persuading some teachers to adopt this novel method. Through the lens of Ertmer’s first- and second-order barriers to change, this paper presents a study began with the need to understand barriers to the adoption of flipped classroom in Hong Kong public secondary schools. Data collected from a questionnaire with open-ended opinion polls for secondary teachers in Hong Kong and their accompanying comments and feedback revealed that both first-order and second-order barriers were hindering their adoption of flipped classrooms into their teaching practices. While the first-order barriers are being resolved with school and government initiatives, a programme of professional development for teachers, as one of the most tangible approaches to capacity building, was provided throughout 2014–2015 school-year as a strategic intervention. Based on the feedback received, some attributes of effective teachers’ capacity building are discussed, and a set of recommendations for catalysing teachers embracement of flipped classroom are given.

## Introduction

Teachers frequently encounter the problem of having too little allotted class time to complete required tasks, but thanks to developments in information and communication technology (ICT), they can use class time more efficiently by incorporating novel technology-based methods in their teaching, in particular, by making material that would traditionally be delivered during a class lecture available for students to access online and utilising class time for discussions and problem-solving activities. This increasingly popular approach, often referred to as the ‘flipped classroom’, has gained the attention of educators and policy-makers. Being a self-titled ‘Smart City’ (Central Policy Unit 2015), Hong Kong has been enthusiastic in implementing flipped classroom teaching and has been encouraging this method in the city’s public K-12 education system through the curriculum guidelines made by the Education Bureau (EDB) (Education Bureau 2014a). However, many teachers in the region have expressed a reluctance to adapt their teaching style to accommodate this technology-based approach. In order to effectively overcome these challenges, it is essential to work with teachers and understand why they may have reservations about the radical change in the structuring of their lessons necessitated by the adoption of flipped classrooms.

## Literature review

### Flipped classroom

Flipped classroom provides a way to alter the traditional pattern of learning and teaching, with faculty posting their class lectures online for students to view so that they can use class time for hands-on application, problem-solving and assessment. In other words, what was formerly schoolwork becomes homework and what was formerly homework becomes schoolwork—hence ‘flipped’.

**Fig. 1:**
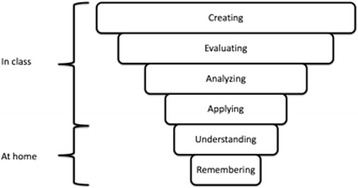
Bloom’s revised taxonomy in a flipped classroom

The flipped classroom approach is not simply about doing homework in class and delivering lecture material digitally to students outside of class. It is, in fact, a pedagogical method that aims to emphasise learning over content delivery by optimising learning and teaching activities (Flipped Learning Network 2014); while in the classroom, the learning and teaching focuses on higher forms of cognitive work (*applying*, *analyzing*, e*valuating* and *creating* in Bloom’s revised taxonomy), and the lower levels (*remembering* and *understanding*) are presented before class through recorded lectures and video (See Fig. 1).

O’Flaherty and Philips (2015), whose research focused on student-centred learning perspectives, gathered that the flipped classroom is superior to traditional methods of learning and teaching, in which lectures are delivered during class and students are asked to engage in supplementary activities outside of class as homework. They identified the following advantages of flipped classrooms:

- Digital learning resources allow students to customise the pace at which they receive new information.
- A reduction in the amount of lecture time enables instructors to increase the amount of two-way communication and relevant problem-solving activities.
- Such discussion and problem-solving promotes active and inquiry-based learning.

In coherence with these advantages, numerous experimental studies have emerged where researchers measured or observed positive changes in students’ *academic performance* (Baepler et al. 2014; Davies et al. 2013; Hung 2015), *motivation* (Enfield 2013; Kim et al. 2014), *engagement* (McLaughlin et al. 2013; McLaughlin et al. 2014), *interaction* (Chen et al. 2014; Love et al. 2014) and *satisfaction* (Mason et al. 2013; Missildine et al. 2013; Schultz et al. 2014). In addition to these benefits, the flipped classroom, with its reusable and easily adaptable materials, has also been shown to be cost-effective (Arnold-Garza 2014).

### First- and second-order barriers to change

Teachers may not be willing to immediately adopt the new method (Fullan 2015), such as the flipped classroom that has significant reliance on ICT meant. They often maintain cautious and would naturally have concerns over that change (Hord et al. 2006).

Ertmer (1999) pointed to first-order and second-order barriers in the implementation of flipped classroom teaching. The former consist of external barriers such as time constraints and a lack of support and resources, while the latter represent the internal challenges posed by teachers’ attitudes, confidence and beliefs.

Ertmer’s studies (Ertmer 1999; 2005) gave priority to addressing second-order barriers, as teachers may be unwilling to adopt technology in the classroom even if all first-order barriers are removed. Fullan noted that instructors have a tendency to rely on their current notions and past experience when faced with the prospect of utilising innovative practices (Fullan 2015).

### Capacity building

The concept of capacity building has been developed in recent years, based on the recognition that it is hard to change existing learning and teaching practices even when there is broad agreement on what the new practices should be (Sugrue 2008). The term has been defined as ‘the development and use of policies, strategies, and actions that increase the collective power or efficacy of whole groups, organisations, or systems to engage in continuous improvement for ongoing student learning’ (Fullan 2005, p.213).

Teachers are gatekeepers of change because they tend to refer to their existing beliefs and prior experiences when attempting to introduce transformative practices into their instruction (Fullan 2015). Their capacity, therefore, needs to be built when new learning and teaching paradigms are being introduced.

## Method

### Rationale and research questions

The rationale behind the study was to understand the concerns over why Hong Kong public school teachers have not been incorporating the flipped classroom model into their teaching and provide interventions that may be able to address these issues.

More specially, this study sought answers to the following research question:

1. *What are the perceived and reported barriers that Hong Kong secondary school teachers encounter if they engage in flipped classroom teaching?*
  The findings of this question were used to assist the research team in understanding teachers’ concerns associated with the adoption of flipped classroom in their schools so that appropriate efforts and interventions could be employed to promote the acceptance and actual use of flipped classrooms.
2. To what extent do measures of capacity building for teachers address Hong Kong secondary school teacher’s barriers to the adoption of flipped classroom?

### Research design

The research team mainly employed a qualitative method (Creswell 2014; Patton 2002) that included a pre-intervention questionnaire distributed to in-service teachers, several post-intervention semi-structured face-to-face interviews and self-reflections of the research team.

The pre-intervention questionnaire, a self-reporting instrument, addressed the significant contingent of variables involved. It began with general background questions and ended with an open-ended opinion poll, in which respondents indicated the three most important barriers they have identified (either through direct experience or through discussion with colleagues) in adopting the flipped classroom method. By applying Ertmer’s classification of first- and second-order barriers (Ertmer 1999; 2005), the research team could better understand the challenges associated with implementing flipped classrooms. Then, the priorities of the capacity building, as interventions, could be decided accordingly. Feedback on the intervention could be obtained through the interviews, which would provide us a basis for putting forward the recommendations.

### Participants

In order to ensure that the pool of respondents was appropriate to the context of the study while also sufficiently diverse, the research team utilised a convenience sampling (Patton 2002). This was done by selecting 210 in-service teachers, from a variety of Hong Kong public secondary schools, including those falling under the categories of aided schools, missionary schools and the Direct Subsidy Scheme. Instructors’ expertise, as well as their prior experience and familiarity with ICT, also varied, and most of them have never tried flipped classroom method to teach. Before the study commenced, all respondents were informed of the confidentiality and anonymity of the collected data, as well as how it would be represented.

### Data collection and analysis

The questionnaires were distributed and collected in May 2014, with the data entered into Microsoft Excel, a spreadsheet software programme.

All responses in Chinese were translated into English. For the sake of consistency, the translation was verified by a separate researcher.

A subsequent thematic analysis (Boyatzis 1998) was carried out, meaning that responses were categorised into first- and second-order barriers.

The study went beyond simply pointing out and investigating barriers to adopting flipped classroom teaching methods. It was important to consider how specific barriers emerge at separate stages of the implementation process, because this information is essential in developing focused strategies for overcoming the hindering factors and facilitating the implementation of flipped classrooms.

## Results

### Preliminary findings

**Fig. 2:**
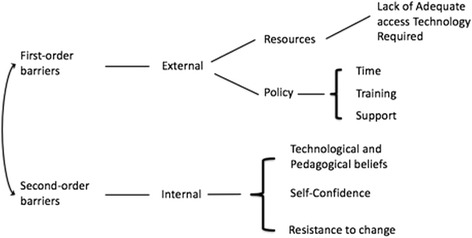
Barriers identified to teachers' adoption of flipped classroom

The results of the questionnaire, based on the thematic analysis of what participating teachers' reported, suggest that teachers involved in adopting flipped classroom teaching encountered first- and second-order barriers simultaneously, as shown in Fig. 2.

### First-order barriers

#### Resources: student accessibility to technology required for flipped lessons

Students’ limited ability to access the technology required for successful flipped classroom teaching was found as a potential inhibiting factor. Some aided schoolteachers expressed that not all of their students have the digital devices necessary (e.g. a laptop or tablet) or a dependable network connection to view course content outside the classroom. Essentially, in Hong Kong, students’ means of accessing the technology required for flipped classroom learning vary according their families’ socioeconomic status. Although the Hong Kong SAR Government has reportedly made progress in the last 3 years—such as the government-covered Wifi 100 Scheme (Education Bureau 2014b), school-offered one-to-one and school-encouraged Bring-Your-Own-Device (BYOD) initiatives (Adams Becker et al. 2016)—to address this digital divide, it seems that not all students have yet benefitted from these efforts.

#### Policy: teacher flipped lesson preparation time, necessary technical training and support

Appropriate policies can both drive or hinder school change and development (De Freitas & Oliver 2005). A considerable number of teachers reported not having supportive policy in place as a significant limiting factor in adopting flipped classrooms.

More specifically, amongst these reported barriers, most respondents (87%) stated that they had struggled with limited time and energy even before they were suggested to implement the flipped classroom model. In other words, the demand on teachers’ time and energy in their existing teaching practice without involving technologies was already great. Although time constraints can become an excuse for some of those teachers who are not technologically inclined to avoid using flipped classrooms, admittedly, without supportive policies that can balance teacher workload, it is unlikely that teachers will have a willingness to adopt flipped classrooms.

Although ICT alone is a facilitator, not a driver for change (Yuen et al. 2003), teachers with limited exposure to ICT would need the availability of technical training, along with just-in-time and continuing support in order to alleviate their technical pressure. However, more than half of the participants reported that their schools do not have the dedicated advisory personnel to guide and support them through their flipped lesson material (such as videos) preparation activities.

### Second-order barriers

The data revealed prominent second-order barriers, which can be classified into issues relating to teachers’ attitudes and self-confidence concerning flipped classroom teaching.

#### Technical and pedagogical beliefs

Most (79%) of the teachers in our study held a prejudicial view that ICT-supported lessons, including flipped classrooms, are not as effective as the conventional teaching paradigm, especially when the student assessment method is a standardised test. Understandably, traditional classroom learning is appealing to these teachers because they understand their roles and what is required of their behaviour and efforts to conduct or achieve a successful learning experience. The introduction of the flipped classroom would change such expectations, as it takes away the comfort zone and presents many unknowns.

#### Self-confidence

Lack of confidence was also a second-order barrier reported that would prevent teachers from using the flipped classroom in their teaching. On the one hand, many teachers who do not consider themselves to be well-skilled in using ICTs reported that they would feel anxious due to their fear of failure and further asserted that ‘losing face’ in front of a class of teenagers who perhaps know more ICT knowledge than they do could be culturally embarrassing. On the other hand, a number of teachers who confidently use ICTs in their classrooms reserved their confidence for pedagogical reasons. More specifically, over half of the teacher respondents (63%) admitted that they lacked confidence in utilising the flipped classroom method because they presumed the flipped classroom model would rely heavily on student self-motivation. Many of those teachers shared the concern that certain groups of students would be less motivated than others, and therefore, end up learning less. Although the participated teachers were from secondary schools, their students can still be considered very young and would perhaps naturally not as self-disciplined as adult learners. Therefore, several teachers further pointed out that expecting their students to keep to a regimented flipped classroom schedule can be daunting, even if they were willing to try. Given the above reasons, a large majority (89%) of the participated teachers reported that they did not have any confidence in adopting the flipped classroom model in their own teaching despite the increasing hype surrounding the unfamiliar approach, and one teacher even claimed the popularity of flipped classroom could be just a ‘dog and pony show’.

#### Attitudes and resistance to change

There were several extreme cases of teachers who were quite resistant towards *all* education-related innovations utilising ICT, including the concept of the flipped classroom itself. They described such tools as ‘new toys’ or novelties meant for reducing monotony in the teaching and learning process. A further investigation found all such sentiments were expressed by teachers at aided schools. A possible explanation might be their relatively low exposure to ICT-supported education innovations due to the limited government findings, which eventually led to their being very conservative even resistant to change.

## Strategic intervention—capacity building for teachers

This study indicated that both first-order and second-order barriers can hinder the adoption of flipped classrooms in Hong Kong public secondary schools. While learning positive results were achieved in some schools regarding the mitigation of first-order barriers, based on the intensity of the barriers identified, we believed our capacity-building measures should focus more on overcoming second-order barriers, although they would flow out of considerations of different perspectives at the macro level, in order to achieve more sustainable outcomes.

One of the most tangible approaches to capacity building for teachers is professional development. However, in many situations, teachers find professional development activities to be ineffective when the programmes fail to connect with teachers’ actual teaching practices (Bradshaw 2002; Wells 2007). Such situations are understandable and need to be addressed sensitively. According to Kubitskey, Fishman and Marx (2003), teachers are more likely to take ownership of a new approach when they actively engage in and reflect on how the approach transforms their own teaching practices.

In line with our findings on the barriers that teachers were experiencing, we became aware that professional development programmes should not be designed merely to familiarise them with new technologies—because ‘adding wings to caterpillars does not create butterflies’ (Marshall 1995). Rather, as ‘teachers have to feel that there is some compelling reason for them to practice differently’ (Elmore 1996, p.24), the first concern to be addressed is justifying why the learning and teaching paradigm shift and pedagogy change (to flipped classroom) are necessary. Teacher appreciation of how these shifts and changes can expose new learning opportunities may serve as the key enablers to make their subjects and disciplines more meaningful to students and more relevant to society. Ultimately, changes in both thought and action can be achieved.

To put the aforementioned measures into action, through linking up with our partners, the research team offered a spectrum of pedagogically focused seminars, sharing sessions and workshops throughout the 2014–2015 school year to showcase innovative flipped classroom practices in different Hong Kong secondary schools and elsewhere. These capacity building activities started with the notion that teachers might get carried away with videos that students are supposed to watch at home, forgetting the whole purpose of flipping the classroom. More specifically, instead of merely training teachers how to use particular software or online systems to create teaching materials for their flipped lessons, participants were offered a number of seminars and sharing sessions from the frontline teachers who are early-adopters or passionate experts of flipped teaching in Hong Kong. The collection of early-adopters’ stories provided a source of inspiration, which encouraged teachers to reflect, recharge and re-imagine the possibilities of learning and teaching and to reflect on their own practice critically. Hands-on workshops that grouped teachers of different disciplines were also provided in order to help teachers to confront pedagogical or administrative challenges that they were likely to encounter in class (for example, what if some students didn’t complete the online tasks before the class)—after all, the main idea behind it is to free up some in-class time for true and engaging learning.

The abovementioned activities were believed not only would raise awareness but also could build confidence by explaining and elaborating the rationale and approaches for adopting flipped classrooms clearly and effectively—thus, addressing secondary barriers such as negative teacher attitudes towards and perceptions of flipped classroom teaching. These activities were complemented the technical workshops we offered for developing teacher’s necessary competency in creating flipped lessons, although the technical aspect of our capacity building was not the key focus.

**Fig. 3:**
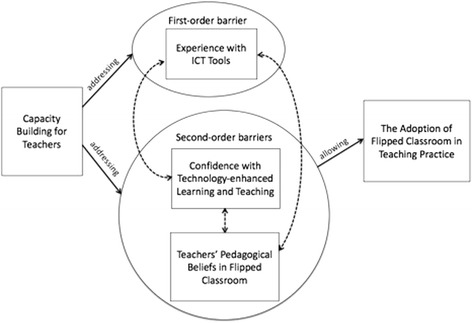
Proposed capacity building model for teachers’ adoption of flipped classroom

The proposed capacity building model for teacher’s adoption of flipped classroom is shown as Fig. 3.

### Post-intervention feedback

It is very satisfying that, throughout the seminars, workshops and training sessions, a growing number of teachers reported being more enthusiastic about adopting flipped classroom in their own teaching practices. According to our administered surveys, which used a 5-point scale, where 1 means very unsatisfied and 5 means very satisfied, teachers evaluated our capacity-building programme with an overall high satisfaction rating of 4.4 (mean). Many reported that the content was ‘interesting and practical’. Most importantly, as the content provided were very ‘pedagogically focused’, ‘case-based’ and ‘context-rich’, over half of the participating teachers (52%) reported that they could directly ‘extract what they learned from those scenarios’ or ‘borrow a thing or two from other’s experiences’ and would be able to apply the knowledge into their own flipped teaching. These assertions may be considered as indicators that teachers’ resistance towards flipped classroom was fading, and their willingness of adopting the model into their own teaching was growing. Teachers’ recognition of the pedagogical values of introducing flipped classroom was also noted as a positive outcome of teacher’s capacity building for overcoming second-order barriers.

## Discussion

### Lessons learned and suggestions

Our successful teachers’ capacity building experience demonstrated that if more teachers come to understand the merits of the flipped classroom approach, they are likely to link such a method to their own teaching practices. In order to be effective, such professional development programmes for flipped classroom need to be more than episodic events but a long-durational process of progression. Along with this spectrum, professional development culture can perhaps be developed with appropriate strategies, such as encouraging the formation of communities of practice (Wenger, 1998; Wenger, 2000; Wenger, McDermott, & Snyder 2002) to deepen teaching staff’s understanding of the intricacies of the flipped classroom paradigm. At the same time, peer mentorship mechanisms that pair leading teachers in the programme with the ones who are less motivated about the approach can be established. Both measures could help to promote positive attitudes amongst teachers regarding flipped classrooms, but yet, neither can be successfully implemented without a sufficient time continuum.

Additionally, effective teachers’ capacity building for flipped classroom also requires a nurturing environment in which teachers can reflect on their own practices in mutually beneficial relationships, and this can also decrease the isolation of classroom practices. Ample opportunities must be provided for teachers to engage in reflective dialogue about their current practices and develop action plans to shape their future practices. Through encouraging teacher autonomy, the free sharing of ideas between teachers and positive peer impact, promising practices in flipped classroom teaching can be fostered. In addition, recognition measures such as rewards and incentives for innovative teachers can prove useful in promoting effective flipped classroom techniques: Teachers can be encouraged to engage in flipped classroom teaching by non-financial rewards such as certificates of merit or recognition.

Teacher capacity building should be based not on a simple desire to improve teachers’ understanding of flipped classroom method alone, but rather flow out of considerations of all possible matters at the macro level. For example, teacher’s capacity building should involve how teachers can prepare students being able to effectively learn a flipped lesson environment. Indeed, although today’s students may be branded as ‘digital natives’ (Prenksy 2001) who are ‘born digital’, as technologies form an integral part of the overwhelming majority of students’ daily routines (Prensky 2011), it has to be acknowledged that many students often lack experience in using technology for learning because they often use it for the purposes of entertainment and communication rather than generating and constructing knowledge (Wang et al. 2014). Students’ lack of familiarity in more self-regulated, active learning environment can hamper teachers’ ability to facilitate a flipped lesson, and thus, need serious consideration.

## Conclusion

Hong Kong secondary school teachers have noted several inhibiting factors in the implementation of flipped classroom teaching. These issues can be classified according to first- and second-order barriers, which involve external challenges (e.g. lack of time, support or resources) and internal challenges (e.g. attitude, confidence and beliefs), respectively. While our analysis identified the concurrent existence of first- and second-order barriers to harnessing the flipped classroom paradigm in teaching, it seems that addressing the second-order challenges might be a greater priority when taking measures to mitigate these problems. With a rethinking of teachers’ capacity building in the form of professional development, a 1-year-long programme was tailored that focused on concerns beyond the development of simple technological know-how. Feedback suggested the research team was on the right direction, and lessons were learned throughout the process. It is hoped that these lessons can serve as ‘food for thought’ for school leaders and policymakers in planning capacity building for flipped classroom in their own contexts. With the concerted effort of both teachers and school leaders and policymakers, the broader implementation of the flipped classroom paradigm may be achieved in Hong Kong.
